# Supplementary material for: AMICI: high-performance sensitivity analysis for large ordinary differential equation models
Source: Bioinformatics. 2021 Apr 2;37(20):3676–7. doi: 10.1093/bioinformatics/btab227 (PMC8545331; doi:10.1093/bioinformatics/btab227)
Supplement: btab227_Supplementary_Data [file btab227_supplementary_data.pdf]

Supplementary Information to AMICI: High-Performance Sensitivity Analysis for Large Ordinary Differential Equation Models

Contents

1 Benchmarking **1**
- 1.1 Benchmarking with PySB 1
- 1.2 Benchmarking simulation of SBML models with COPASI 2

2 Feature comparison of Systems Biology ODE simulation tools **3**

1 Benchmarking

1.1 Benchmarking with PySB

To compare simulation performance with PySB, we evaluated simulation run times for the 17 PySB models using the parameter values in the respective PySB examples. Absolute and relative tolerances were both set to 10^8, otherwise default options were used for simulation in AMICI (`v0.11.13`) as well as PySB (commit `6a3e9a5`). Reported simulation times are averaged over 100 repetitions. The script that was used to generate this figure was added to the AMICI repository and is available `here`.

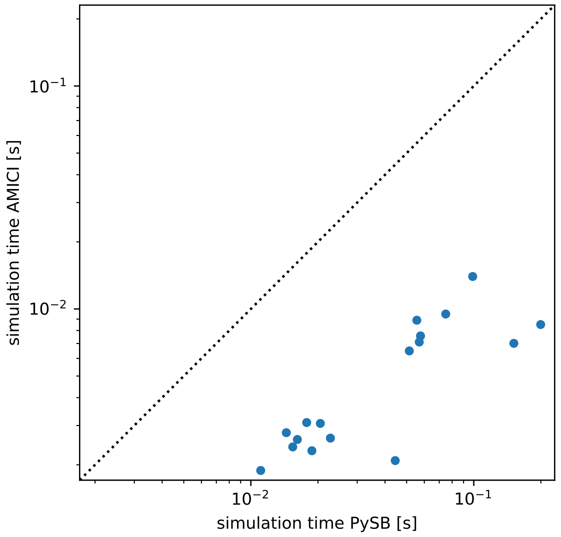

1.2 Benchmarking simulation of SBML models with COPASI

We furthermore compared simulation times of ODE systems in AMICI and COPASI for three SBML models taken from different publications. Since gradient-based parameter estimation is a typical application of AMICI and COPASI, we compared the computation time for evaluating the objective function and its gradient for these models, given the measurement data which was available in the corresponding publications. As integration error tolerances, the default settings of COPASI were used for both toolboxes, i.e., a tolerance of 10^{-12} fot the absolute error and of 10^{-6} for the relative error.

Since importing an SBML model in AMICI includes symbolic calculations, generation of model-specific C++ files, and their compilation, model import is more time-consuming with AMICI than with COPASI. However, objective function and in particular gradient evaluation is faster in AMICI and scales better to large model sizes. We therefore calculated the break-even point, i.e., after how many gradient evaluations in parameter estimation the higher import time with AMICI is amortised. A typical parameter estimation problem requires in the order of (some) ten thousands to some millions of gradient evaluations. Beyond speeding up parameter estimation, AMICI computes gradients via semi-analytical sensitivity analysis, yielding numerically substantially more accurate results (Fröhlich *et al.*, 2017).

Table 1: Computation time comparison for COPASI and AMICI. All models were taken from peer-reviewed publications, only one experimental condition of the published datasets was used for objective function and gradient evaluation at reported parameter values, respectively.

**Model publication**	Crauste et al.	Lucarelli et al.	Froehlich et al.
**No. of species**	5	33	1396
**No. of parameters**	12	72	4233
**CPU time likelihood evaluation [ms]**			
**AMICI**	0.50	0.69	168.39
**COPASI**	0.87	2.50	35089.28
**COPASI / AMICI**	1.74	3.62	208
**CPU time gradient evaluation [ms]**			
**AMICI**	9.71	27.29	1264.91
**COPASI**	11.33	183.04	$1.49{\cdot}10^{8}$
**COPASI / AMICI**	1.17	6.71	97600
**CPU time model import [s]**			
**AMICI**	27.14	39.73	1521.40
**COPASI**	0.23	0.33	100.20
**No. of gradient evaluations to break-even point**			
	16925	253	1

2 Feature comparison of Systems Biology ODE simulation tools

Table 1: Feature comparison of prominent simulation tools supporting model definition in high level formats in the field of systems biology. The presented list of features is non-exhaustive.

	**AMICI**	**COPASI**	**D2D**	**libroadrunner**	**BIOPARKIN**	**SimBiology**	**SloppyCell**
**Language**	Python/C++/MATLAB	stand-alone GUI (C++)	MATLAB	Python, C++, C	stand-alone GUI (C++)	MATLAB	Python/C
**Models**							
- ODE	✓	✓	✓	✓	✓	✓	✓
- ODE with events	✓	✓	✓[1]	✓	✓	✓	✓
- DAE	☐	☐	☐	✓	☐	✓	☐
- DAE with events	☐	☐	☐	✓	☐	✓	☐
**Model definition**							
- SBML	✓	✓	✓	✓	✓	✓	✓
- PySB	✓	☐	☐	☐	☐	☐	☐
**Simulation**							
- time resolved	✓	✓	✓	✓	✓	✓	✓
- steady-state	✓	✓	✓	✓	☐	☐	✓
**Forward sensitivities**							
- time resolved	✓	✓[2]	✓	☐	✓	✓	✓
- steady-state	✓	✓[2]	✓	☐	☐	☐	☐
**Adjoint sensitivities**							
- time resolved	✓	☐	☐	☐	☐	☐	☐
- steady-state	✓	☐	☐	☐	☐	☐	☐
**Linear solvers**							
- dense	✓	✓	✓	✓	☐	✓	✓
- sparse	✓	☐	✓	☐	☐	✓	☐
- iterative	✓	☐	☐	☐	✓	☐	☐
**Year of latest release (latest activity)**	2021	2021	2017 (2021 GitHub commits)	2020 (2021 GitHub commits)	2012 (2015 GitHub commits)	2020	2005 (2017 SourceForge commits)
**Open source**	✓	✓	✓	✓	✓	☐	✓
**Licence model**	BSD-3-Clause	Artistic-2.0	custom	Apache-2.0	LGPL-3.0	commercial	BSD-3-Clause

[1] State-dependent events are not supported
[2] Only finite differences, which is numerically problematic.

References

Crauste, F., Mafille, J., Boucinha, L., Djebali, S., Gandrillon, O., Marvel, J., and Arpin, C. (2017). Identification of nascent memory CD8 T cells and modeling of their ontogeny. *Cell Systems*, **4**(3), 306–317.

Fröhlich, F. *et al.* (2018). Efficient parameter estimation enables the prediction of drug response using a mechanistic pan-cancer pathway model. *Cell Syst.*, **7**(6), 567–579.e6.

Fröhlich, F., Kaltenbacher, B., Theis, F. J., and Hasenauer, J. (2017). Scalable parameter estimation for genome-scale biochemical reaction networks. *PLoS Comput Biol*, **13**(1), e1005331.

Lucarelli, P., Schilling, M., Kreutz, C., Vlasov, A., Boehm, M. E., Iwamoto, N., Steiert, B., Lattermann, S., Wšch, M., Stepath, M., Matter, M. S., Heikenwälder, M., Hoffmann, K., Deharde, D., Damm, G., Seehofer, D., Muciek, M., Gretz, Norbert Lehmann, W. D., Timmer, J., and Klingmüller, U. (2018). Resolving the combinatorial complexity of smad protein complex formation and its link to gene expression. *Cell Syst.*, **6**(1), 75–89.
